# P-1071. In Vitro Susceptibility of the syphilis spirochete, *Treponema pallidum*, to Linezolid

**DOI:** 10.1093/ofid/ofae631.1259

**Published:** 2025-01-29

**Authors:** Chrysovalantis Stafylis, Bridget D De Lay, Steven Norris, Ryan M Nguyen, Kelika Konda, Michael Reyes-Diaz, Jeffrey Klausner, Diane Edmondson

**Affiliations:** University of Southern California, Los Angeles, California; University of Texas Health Science Center at Houston, Houston, Texas; McGovern Medical School, UT Health, Houston, Texas; University of Southern California, Los Angeles, California; Universidad Peruana Cayetano Heredia, Lima, Lima, Peru; Universidad Peruana Cayetano Heredia, Lima, Lima, Peru; Keck School of Medicine of USC, Los Angeles, California; McGovern Medical School, UT Health, Houston, Texas

## Abstract

**Background:**

Benzathine penicillin is the antimicrobial treatment of choice for syphilis, but worldwide shortages have caused disruptions in patient care. Therefore, the is a need for new antimicrobial compounds that are effective at curing *Treponema pallidum* subsp. *pallidum* (Tp) infections that are not subject to the same supply chain issues currently effecting benzathine penicillin availability. To this end, we tested the in vitro susceptibility of four different strains of Tp (Nichols, Mexico A, UW231B, and UW249B) to linezolid.

**Methods:**

There are two genetically distinct subgroups of Tp: the Nichols group and the SS14 group. The SS14 group is currently predominant worldwide, so we tested three different strains from this group (Mexico A, UW231B, and UW249B). Tp cultured in TpCM2 medium were exposed to linezolid concentrations ranging from 0 to 4 µg/mL for 1 week, then the number of Tp per culture and motility were assessed by darkfield microscopy.

**Results:**

Multiplication was completely inhibited (fold increase ≤1) in cultures treated with 0.5 - 4 µg/mL linezolid and motility was significantly decreased. In vitro MICs for linezolid ranged from 0.32 to 0.36 µg/mL in the four strains tested, with no difference between the Nichols strain (0.32 µg/mL) and the SS14 subgroup strains (0.34 - 0.36 µg/mL). The MIC range determined in vitro is compatible with the blood serum ranges reported in patients given therapeutic doses of linezolid.

**Conclusion:**

Further studies are ongoing to determine if linezolid is an effective antimicrobial compound for the treatment of syphilis.

**Disclosures:**

**Jeffrey Klausner, MD MPH**, Direct Diagnostics: Advisor/Consultant

